# Protein A/G-based enzyme-linked immunosorbent assay for detection of anti-*Pythium insidiosum* antibodies in human and animal subjects

**DOI:** 10.1186/s13104-020-04981-y

**Published:** 2020-03-06

**Authors:** Chalisa Jaturapaktrarak, Penpan Payattikul, Tassanee Lohnoo, Yothin Kumsang, Aree Laikul, Watcharapol Pathomsakulwong, Chompoonek Yurayart, Walaiporn Tonpitak, Theerapong Krajaejun

**Affiliations:** 1 Research Center, Faculty of Medicine, Ramathibodi Hospital, Mahidol University, Bangkok, Thailand; 2 Molecular Medicine Program, Multidisciplinary Unit, Faculty of Science, Mahidol University, Bangkok, Thailand; 3 Department of Large Animal and Wildlife Clinical Sciences, Faculty of Veterinary Medicine, Kasetsart University, Nakhon Pathom, Thailand; 4 Equine Clinic, Kasetsart University Veterinary Teaching Hospital, Nakhon Pathom, Thailand; 5 Department of Microbiology and Immunology, Faculty of Veterinary Medicine, Kasetsart University, Bangkok, Thailand; 6 Department of Microbiology, Faculty of Veterinary Medicine, Mahanakorn University of Technology, Bangkok, Thailand; 7 Department of Pathology, Faculty of Medicine, Ramathibodi Hospital, Mahidol University, Bangkok, Thailand

**Keywords:** *Pythium insidiosum*, Pythiosis, Immunodiagnosis, ELISA, Protein A/G

## Abstract

**Objectives:**

Pythiosis is a deadly infectious disease caused by *Pythium insidiosum.* Reports of both human and animal pythiosis are on the rise worldwide. Prognosis of the pythiosis patients relies on early diagnosis and prompt treatment. There are needs for an immunodiagnostic test that can detect the disease in both humans and animals. This study aims at reporting an optimized protocol for the development of a protein A/G-based enzyme-linked immunosorbent assay (ELISA) for the detection of anti-*P. insidiosum* antibody in multiple host species.

**Results:**

A total of 25 pythiosis and 50 control sera, obtained from humans, horses, dogs, cats, and cows, were recruited for the assay development. With a proper ELISA cutoff point, all pythiosis sera can ultimately be distinguished from the control sera. The successfully-developed protein A/G-based ELISA can detect the anti-*P. insidiosum* antibodies in serum samples of both humans and animals. It is a versatile, feasible-to-develop, and functional immunodiagnostic assay for pythiosis.

## Introduction

Pythiosis is a deadly infectious disease caused by the pathogenic oomycete *Pythium insidiosum* [1, 2]. The disease affects humans and animals, especially horses and dogs. Geographic distribution of pythiosis covers the tropical and subtropical countries [2]. Pythiosis demonstrates high rates of morbidity and mortality [3]. Most of the affected individuals lose an infected organ, and many patients die from the disease. Prognosis of the pythiosis patients relies on early diagnosis and prompt treatment. Establishing the definitive diagnosis requires a reliable diagnostic method, i.e., organism isolation and identification, immunodiagnostic test, molecular assay, and proteomic analysis [1, 2, 4–13].

Immunodiagnostic tests for pythiosis have gained popularity due to simplicity and short turnaround time. Various immunodiagnostic methods, such as immunodiffusion (ID), enzyme-linked immunosorbent assay (ELISA), hemagglutination (HA), immunochromatographic test (ICT), have been developed to facilitate the diagnosis of pythiosis [10–20]. Each immunodiagnostic test has some advantages/disadvantages over the others. For example, ID and HA detect the antibodies in both humans and animals but possess limited sensitivity. ELISA is a multi-step assay but exhibits relatively-high diagnostic performance. ICT is a rapid and user-friendly assay, but the development of this test is complicated. Because reports of both human and animal pythiosis are on the rise worldwide, there are needs for an immunodiagnostic test that can detect the disease in a broad range of host species. Due to the lack of a versatile, feasible-to-develop, and functional immunodiagnostic assay, the current study aims at reporting an optimized protocol to develop an ELISA for the detection of anti-*P. insidiosum* antibodies in humans and other animals. The assay relied on the use of protein A/G, which is a molecule that can bind the immunoglobulins from various animal species [16].

## Main text

We extracted proteins from the *P. insidiosum* strain Pi-S [21, 22], using the established method [17, 23]. In short, the organism was maintained on Sabouraud dextrose agar [10 mg/ml peptone (Becton, Dickinson and Company, Maryland, USA), 20 mg/ml dextose (HiMedia Laboratories, Mumbai, India) and 15 mg/ml agar (Becton, Dickinson and Company, Maryland, USA)]. Ten excised agar cubes (~ 0.5 cm^3^) with *P. insidiosum* colony were incubated with shaking (150 rpm) at 37 °C for 10 days in 100 ml of Sabouraud dextrose broth. The growing organism was removed from the cultured broth by consecutive filtrations through a filter paper (Whatman No.1, GE healthcare, Belfast, UK) and a 0.22-μm-pore-size Durapore membrane (Merck Millipore, Cork, Ireland). The filtrated broth was concentrated by centrifugation (10,000×*g*) with an Amicon Ultra 15 M tube (Merck Millipore, Cork, Ireland). The concentrated broth, now called culture filtrate antigen (CFA), was stored at − 30 °C.

We developed a multiple host-specific ELISA by modifying the human-specific ELISA protocols of Chareonsirisuthigul et al. [15] and Lohnoo et al. [23]. In brief, a 96-well flat-bottom polystyrene plate (Corning, New York, USA) was coated overnight at 4 °C with 100 µl/well of CFA (5 µg/ml) in 0.1 M carbonate buffer pH 9.6 [0.2 M Na_2_CO_3_ and 0.2 M NaHCO_3_ (Merck, Darmstadt, Germany)] and 1.5% NaCl (Merck, Darmstadt, Germany). The plate was washed 4 times with the washing buffer [phosphate-buffered saline pH 7.4 (PBS); 137 mM NaCl, 2.7 mM KCl, 10 mM Na_2_HPO_4_, and 1.76 mM KH_2_PO (Merck, Darmstadt, Germany) with 0.05% Tween-20 (Calbiochem, California, USA)], blocked with 250 µl of 0.5% bovine serum albumin (Merck, Darmstadt, Germany) in PBS at 37 °C for 60 min, and washed 4 times again. Each serum (1 µl) was added to 9 ul of PBS. Then, the diluted serum (5 µl) was mixed with 795 µl of PBS. The resulting serum sample (1:1600 in PBS; 100 µl) was added to each well and incubated at 37 °C for 60 min. After washing (as above), 100 µl of protein A/G conjugated with peroxidase (Bio-Rad Laboratories, California, USA) (1:100,000 in PBS) was added to each well and stored at 37 °C for 60 min. The ELISA plate was washed once again. Chromogenic substrate [3,3′,5,5′-Tetramethylbenzidine and H_2_O_2_ (Thermo Scientific, Rockford, USA)] was applied to each well (100 µl/well) and incubated at room temperature for 3 min in a dark chamber. Sulfuric acid (0.5 N; 100 µl/well) was used to stop the enzymatic reaction. Optical density (OD) of each serum sample was measured at the 450-nm wavelength.

**Fig. 1:**
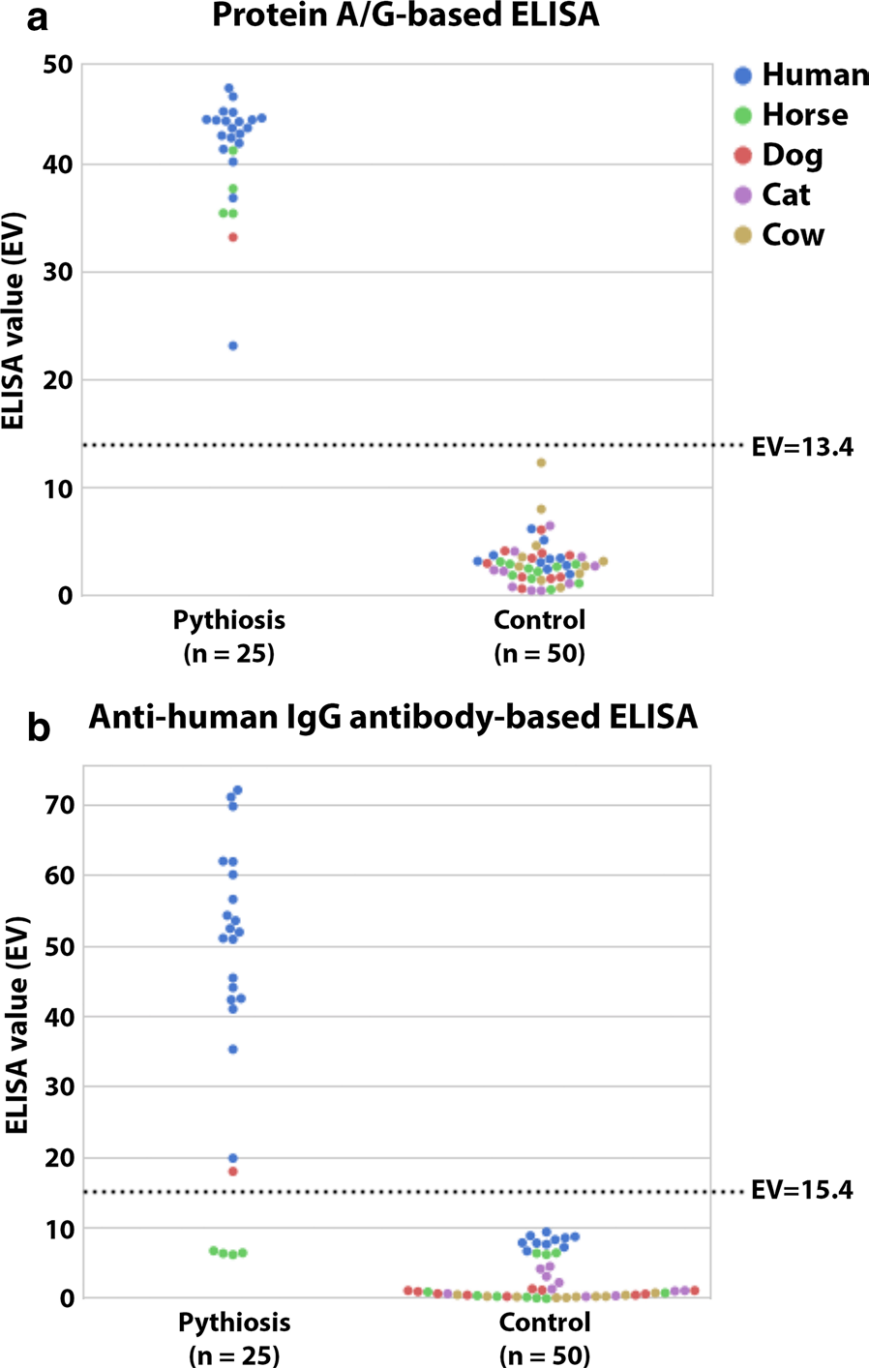
Dot plot of the ELISA values (EV) of pythiosis (n = 25) and control (n = 50) serum samples examined by **a** the protein A/G-based and **b** the anti-human IgG antibody-based ELISAs. Different colors indicate the EVs of sera from different animal species. Dashed lines represent the optimal ELISA cutoff points (see Table 1)

**Fig. 2:**
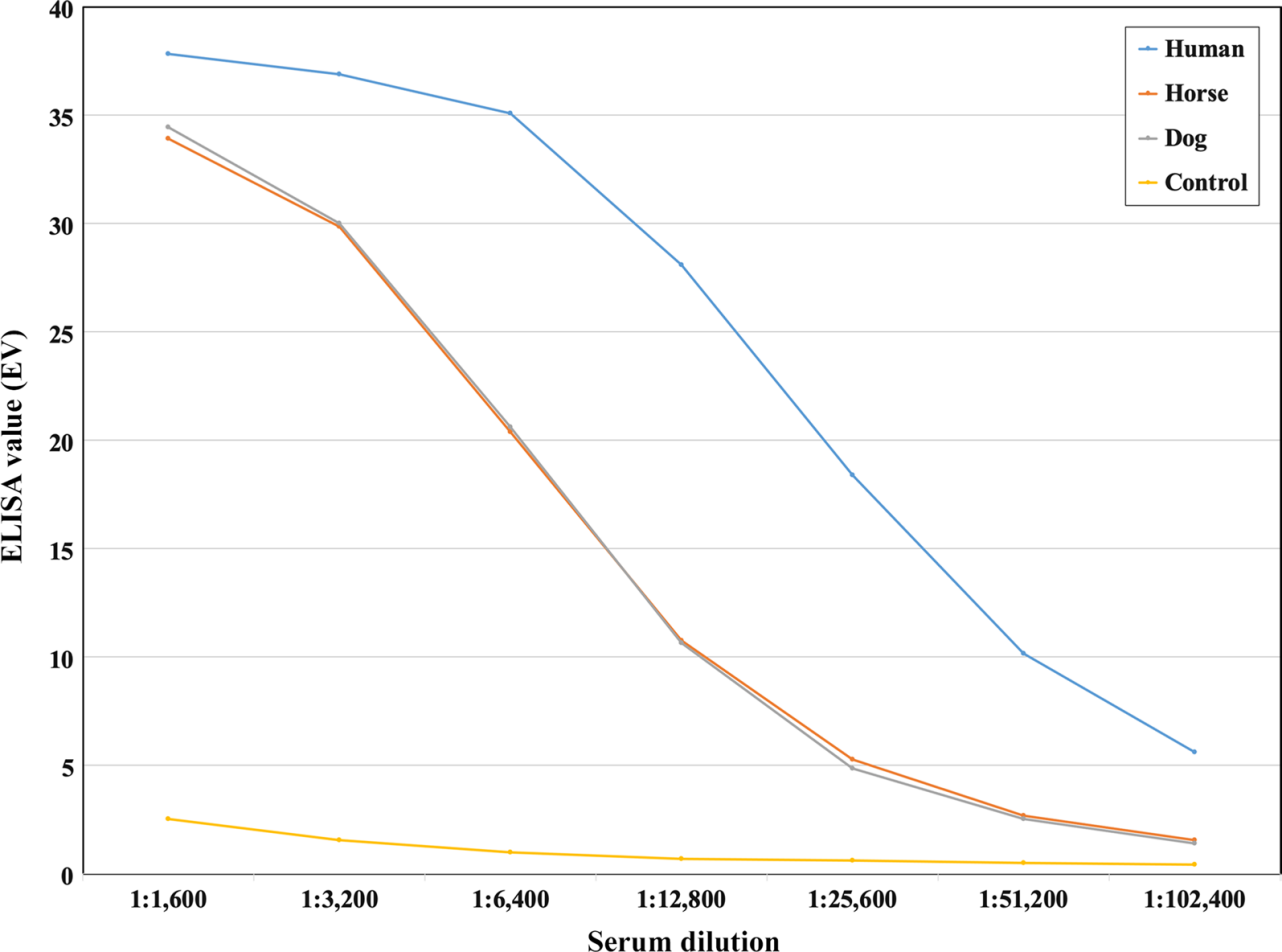
ELISA values (Y-axis) of serial twofold dilutions (from 1:1600 to 1:102,400; X-axis) of a serum sample from a human (blue), a horse (orange), and a dog (gray) with pythiosis, and a healthy individual (served as a control; yellow). The working serum dilution for protein A/G-based ELISA testing is 1:1600 (see text for details)

A total of 25 serum samples were derived from 20 Thai patients, 4 horses, and a dog with the clinical features of pythiosis [1–3], together with either successful detection of *P. insidiosum* by culture and molecular analysis [6, 24–26] or serum anti-*P. insidiosum* antibodies by ICT [16]. Fifty control (negative) sera were obtained from 10 each of individuals, horses, dogs, cats, and cows with no clinical sign of pythiosis in the absence of serum anti-*P. insidiosum* antibodies by ICT. Positive (from a known pythiosis patient) and negative (from a healthy blood donor) control sera were analyzed in parallel. All serum samples were leftover specimens obtained from the Faculty of Medicine, Ramathibodi Hospital, Mahidol University, and the Faculty of Veterinary Medicine, Kasetsart University. Each serum sample was tested in duplicate by the protein A/G-based ELISA. ELISA value (EV) was an OD value of a serum sample divided by an OD value of the negative control serum. ELISA cutoff value was derived from the mean EV of the control sera plus 2, 3, 4, or 5 standard deviations (SD). Diagnostic performance (i.e., specificity, sensitivity, and accuracy) were calculated as described previously [15] (Table 1). Distribution of EVs (representing a detectable level of anti-*P. insidiosum* antibodies) of the pythiosis (n = 25) and control (n = 50) sera from both humans and animals was shown in Fig. 1a. Mean EV of the pythiosis sera was 41.0 (range 23.1–46.9), whereas that of the control sera was 3.0 (range 0.4–12.2). The “mean EV + 5SDs” cutoff point (EV, 13.4) provided the best sensitivity, specificity, and accuracy for the detection of the anti-*P. insidiosum* antibodies (Table 1). Furthermore, protein A/G-based ELISA was performed using serial twofold dilutions (from 1:1600 to 1:102,400) of a serum sample from a human, a horse, and a dog with pythiosis, and a healthy individual (control). The serum dilutions and the obtained EVs of each animal species showed some degree of a linear relationship (i.e., the higher dilution, the lower EV) (Fig. 2). The result suggests that the protein A/G-based ELISA can quantitate an anti-*P. insidiosum* antibody level for monitoring the clinical course of pythiosis.

**Table 1:** Correlation between the cutoff points and the diagnostic performance (i.e., sensitivity, specificity, and accuracy) of the protein A/G-based and anti-human IgG antibody-based ELISAs for detection of the anti-*P. insidiosum* antibodies in serum samples

Cutoff point^a^	Protein A/G-based ELISA				Anti-human IgG antibody-based ELISA			
	Cutoff value (EV)^b^	Sensitivity^c^	Specificity^d^	Accuracy^e^	Cutoff value (EV)	Sensitivity	Specificity	Accuracy
Mean EV + 5SDs	13.4^f^	100.0	100.0	100.0	18.6	80.0	100.0	93.3
Mean EV + 4SDs	11.3	100.0	98.0	98.7	15.4^g^	84.0	100.0	94.7
Mean EV + 3SDs	9.3	100.0	98.0	98.7	12.2	84.0	100.0	94.7
Mean EV + 2SDs	7.2	100.0	96.0	97.3	9.0	84.0	98.0	93.3

^a^Cutoff point = mean EV of the control sera plus 2, 3, 4 or 5 SDs

^b^ELISA value (EV) = OD value of a serum sample/OD value of the negative control serum

^c^Sensitivity (%) = true positive samples/(true positive + false negative samples) × 100

^d^Specificity (%) = true negative samples/(true negative + false positive samples) × 100

^e^Accuracy (%) = (true positive + true negative samples)/all samples × 100

^f^Optimal cutoff point of the protein A/G-based ELISA

^g^Optimal cutoff point of the anti-human IgG antibody-based ELISA

All serum samples were also tested in duplicate by the established human-specific ELISA employing goat anti-human IgG antibody conjugated with peroxidase (Bio-Rad Laboratories, California, USA) [15, 23]. The anti-human IgG antibody-based ELISA with an optimal cutoff point (mean EV + 4SDs, 15.4; Table 1; Fig. 1b) did not detect the anti-*P. insidiosum* antibodies in any control sera tested (EV range 0.0–9.3). This assay can detect the anti-*P. insidiosum* antibodies only in the sera from humans (EV range 19.8–72.0) and a dog (at a relatively-low EV, 17.9), but not horses (EV range 6.1–6.6), with pythiosis. The result indicates that the goat anti-human IgG antibody could strongly bind human antibodies and slightly cross-react with dog antibodies. Based on the same serum samples from humans and animals, the anti-human IgG antibody-based ELISA showed equivalent detection specificity (100%), but markedly-lower detection sensitivity (84% vs. 100%), when compared with the protein A/G-based ELISA (Table 1).

With a proper cutoff point, all pythiosis sera can ultimately be distinguished from the control sera by the protein A/G-based ELISA (Table 1; Fig. 1a). Unlike the species-specific ELISAs established here (Table 1; Fig. 1b) and by other investigators [10–15], the protein A/G-based ELISA is capable of detecting not only the anti-*P. insidiosum* antibodies in a single animal species, but also in humans and various animals (i.e., horses and dogs) those are the most affected hosts of pythiosis. A key feature of our ELISA is the use of protein A/G (rather than a species-specific IgG) as a conjugate, which can bind the anti-*P. insidiosum* immunoglobulins from humans and other animal species. Recently, Intaramat et al. have developed the protein A/G-based ICT, as a rapid and user-friendly assay for diagnosis of pythiosis in humans and animals [16]. They performed a side-by-side performance comparison of their ICT [16] and the species-specific ELISA [11, 13, 15] and showed that ICT has equivalent specificity but relatively-lower sensitivity. One major obstacle for clinical use of ICT is the assay production, which is very complicated and requires a skilled developer and some special reagents that are not available in general clinical laboratories. As opposed to ICT, although ELISA is a multi-step assay and has a longer turnaround time, the production of ELISA is more feasible and requires widely-available reagents. In conclusion, protein A/G-based ELISA has been successfully developed for immunodiagnosis of both human and animal pythiosis. It is a versatile, feasible-to-develop, and functional immunodiagnostic assay.

## Limitations

The newly-developed protein A/G-based ELISA overcame the limitation of the previously-established ELISAs [10–15], as it can detect the serum anti-*P. insidiosum* antibodies in multiple host species, including the most affected hosts: humans, horses, and dogs. Other investigators could adopt our protocol to develop their own in-house ELISA. However, there are several limitations in association with the development and performance evaluation of the assay that need to be addressed:

1. The *P. insidiosum* strain Pi-S was used to prepare the coating antigen. Although this strain has been studied extensively [21, 22, 27], it is not available in the ATCC and CBS-KNAW culture collection centers. Alternatively, any other *P. insidiosum* strains could be used to prepare the coating antigen as well.
2. In the evaluation of our protein A/G-based ELISA, only pythiosis serum samples from 4 horses and a dog were tested, due to the low prevalence of the disease in animals in Thailand.
3. The current study focuses on reporting an optimized protocol for the development of protein A/G-based ELISA. Thus, the diagnostic performance (i.e., detection sensitivity and specificity) of the assay was not extensively investigated, using a variety of serum samples from humans and animals with pythiosis and other diseases.
4. Like the previously-established ELISAs, one extra step to perform the assay is to make a serum dilution (our assay requires 1:1600 dilution). This step is necessary for reducing the background ELISA signals from the bindings of non-specific antibodies and *P. insidiosum*’s crude protein extract. In the future, if a purified *P. insidiosum*-specific protein is available for coating the ELISA plate, then an undiluted serum could be directly tested.
5. Pythiosis patients usually present with a chronic infection, as the mean duration of symptoms before receiving medical care is several months [3]. Direct detection of *P. insidiosum* is an indicator of an active infection. Isolation of *P. insidiosum* from a clinical specimen is difficult and challenging. Antibody detection is an alternative method, providing indirect diagnostic evidence for the *P. insidiosum* infection. Like the other established immunodiagnostic tests for pythiosis, the protein A/G-based ELISA cannot selectively detect an acute-phase infectious marker, such as IgM. Therefore, it cannot directly differentiate between acute, chronic, and previous *P. insidiosum* infections. However, in a patient with the typical features of pythiosis, an elevated or high level of the serum anti-*P. insidiosum* antibodies detected by ELISA suggests an active infection.

## Data Availability

The *P. insidiosum* strain Pi-S (for crude protein preparation) is available upon request.

## Abbreviations

CFA: Culture filtrate antigen
ELISA: Enzyme-linked immunosorbent assay
EV: ELISA value
HA: Hemagglutination
ICT: Immunochromatographic test
ID: Immunodiffusion
OD: Optical density
PBS: Phosphate-buffered saline
SD: Standard deviations
